# Antioxidant and Antibacterial Activity of Mexican Oregano Essential Oil, Extracted from Plants Occurring Naturally in Semiarid Areas and Cultivated in the Field and Greenhouse in Northern Mexico

**DOI:** 10.3390/molecules28186547

**Published:** 2023-09-09

**Authors:** Ruben I. Marin-Tinoco, Angie Tatiana Ortega-Ramírez, Maricela Esteban-Mendez, Oscar Silva-Marrufo, Laura E. Barragan-Ledesma, Luis M. Valenzuela-Núñez, Edwin A. Briceño-Contreras, Maria A. Sariñana-Navarrete, Abelardo Camacho-Luis, Cayetano Navarrete-Molina

**Affiliations:** 1 Faculty of Medicine and Nutrition, Juarez University of the State of Durango, Calle Constitucion 404, Zona Centro, Durango 34100, Durango, Mexico; 2 Management, Environment and Sustainability Research Group, Chemical and Environmental Engineering Department, Universidad de America, Bogotá 110311, Colombia; 3 Interdisciplinary Research Center for Integral Regional Development Unit Durango, National Polytechnic Institute, Sigma 119, Fraccionamiento 20 de Noviembre II, Durango 34220, Durango, Mexico; 4 Department of Engineering, National Technological of Mexico, Technological Institute of the Guadiana Valley, Carretera Durango-México, Km. 22.5, Ejido Villa Montemorelos, Durango 34371, Durango, Mexico; 5 Faculty of Biological Sciences, Juarez University of the State of Durango, Gomez Palacio 35010, Durango, Mexico; 6 Department of Chemical Area Environmental Technology, Technological University of Rodeo, Carretera Panamericana, Km. 159.4, Col. ETA, Rodeo 37560, Durango, Mexico

**Keywords:** oregano, bacteria, microorganisms, carvacrol, thymol, antimicrobials, wild plants

## Abstract

In recent years, the determination of the antioxidant and antibacterial activity of essential oils in wild plants, such as Mexican oregano (*Lippia graveolens* Kunth), has become increasingly important. The objective was to compare the antioxidant and antibacterial activity of Mexican oregano essential oil obtained from plants occurring naturally in semiarid areas (Wild1 and Wild2), and those cultivated in the field (CField) and greenhouse (CGreenhouse) in northern Mexico. The Mexican oregano essential oil extraction was performed using the hydrodistillation method, the antioxidant activity was determined using the ABTS method, and the antibacterial activity was assessed through bioassays under the microwell method at nine different concentrations. The aim was to determine the diameter of the inhibition zone and, consequently, understand the sensitivity level for four bacterial species. The results revealed an antioxidant activity ranging from 90% to 94% at the sampling sites, with Wild1 standing out for having the highest average antioxidant activity values. Likewise, six out of the nine concentrations analyzed showed some degree of sensitivity for all the sampling sites. In this regard, the 25 µL mL^−1^ concentration showed the highest diameter of inhibition zone values, highlighting the Wild2 site, which showed an average diameter greater than 30 mm for the four bacteria tested. Only in the case of *S. typhi* did the CGreenhouse site surpass the Wild2, with an average diameter of the inhibition zone of 36.7 mm. These findings contribute to the search for new antioxidant and antibacterial options, addressing the challenges that humanity faces in the quest for opportunities to increase life expectancy.

## 1. Introduction

In the history of humankind, natural vegetation has represented an important source of natural products that have been widely used in cosmetics, food, and traditional medicine [1,2]. In recent years, there has been a special focus on the search for compounds in natural products to preserve foods (antioxidant activity (AoA)) and protect them from microorganisms (antimicrobial activity (AmA)) [3,4,5]. A plant that has been investigated for these characteristics is oregano, which comprises over two dozen different plant species. This aromatic, bushy, deciduous, and perennial species is highly branched and can reach a height of two meters with a foliage coverage diameter of one meter, exhibiting relatively fast growth and a short life cycle [6]. Lawrence [7] and Russo et al. [8] reported that there are four groups of oregano commonly used for culinary purposes: Greek oregano (*Origanum vulgare* spp. *hirtum* (Link) Ietswaart), Spanish oregano (*Coridohymus capitatus* (L.) Hoffmanns & Link), Turkish oregano (*Origanum onites* L.), and Mexican oregano (*Lippia graveolens* Kunth). Additionally, it is widely used in traditional medicine for a variety of ailments, relying on the extraction of its essential oils (EO), which possess recognized AoA and antibacterial activity (AbA) [9,10,11]. In the scientific literature, numerous reports strongly associate the chemical composition of oregano essential oil (OEO) with the antioxidant, antibacterial, antifungal, antiparasitic, and antimicrobial properties in several oregano species, as well as with its commercial applications [1,3,5,9,10,11,12,13,14,15,16,17,18,19,20,21,22,23,24,25,26,27,28]. Consequently, given the wide range of reported properties of OEO, it stands as one of the favorite candidates for the natural production of antioxidants and antimicrobials.

The EOs are mixtures of volatile organic compounds [29] derived from the metabolism of plants [30], which can be obtained from flowers, seeds, leaves, branches, bark, or roots [31]. Their main compounds are terpenoids, alcohols, aldehydes, ketones, esters, and acids, which confer a characteristic aroma on them [32]. The yield and chemical composition of, and the concentration of secondary metabolites present in, OEO can be affected by genetic, geographical, and climatic factors such as photoperiod, altitude, harvest season, growth stage, temperature, hydric stress, soil composition [2,9,11,33,34,35], origin, extraction method, and the plant part from which the essential oil is extracted [23]. This applies even when the samples come from the same region [36]. The composition of OEO includes terpenes, mainly thymol and carvacrol [14]. These OEOs are generally recognized as safe by the Food and Drug Administration [37], and as a food additive by the European Union [38]. This recognition is due to the characteristics of these OEOs, such as rapid action, absence of residues, difficulty in developing drug resistance, low phytotoxicity [39], and safety for mammals [39,40].

Consequently, in recent years, food preservation systems have sought alternative substances to preserve the final products and protect them from oxidation and microorganisms [3]. However, the use of OEO as a food additive is limited, probably due to its intense aroma, which could result in unacceptable organoleptic properties in food products. This limitation could be minimized by combining it with other natural compounds, achieving additional improvements in the beneficial antioxidant and antimicrobial properties of OEO [38]. Furthermore, in the manufacturing of primary packaging, the volatile nature of the main compounds in OEO should be considered [41]. Despite the publication of scientific articles highlighting the benefits of OEO, this research field remains wide open due to the considerable variability reported in the characteristics of OEOs, even when originating from the same region.

Investigating such variability is of vital importance, in order to develop further improvements, beneficial to human health, in food production and preservation. This production should consider a sustainable and holistic vision aligned with the objectives of sustainable development and all productive industries. This will facilitate the promotion of strategies aimed at the sustainable use of natural resources [42,43,44,45]. Based on the aforementioned, the present study aimed to determine the chemical composition in the OEO from plants occurring naturally in semiarid areas, and from those cultivated in the field and greenhouses in northern Mexico, and evaluate its AoA and AbA. Taking into account the attributes of OEO, the hypothesis was formulated that Mexican oregano essential oil (MOEO) produced by plants occurring naturally in semiarid areas exhibits higher AoA and AbA than MOEO produced by plants cultivated in the field and greenhouse.

## 2. Results

### 2.1. Chromatographic Analysis of the Chemical Composition of MEOE

The average yield obtained during the MEOE extraction process was 30.25 mL kg^−1^ DW^−1^. The calculated density was 920 ± 0.98 µg mL^−1^. The means of the results obtained in the chromatographic analyses for the main reported components of the EOE are presented in Table 1a. In this regard, the combined gas chromatography–mass spectrophotometry analysis demonstrated that the five main chemical components in the analyzed samples were Carvacrol (Phenol, 2-methyl-5-(1-methylethyl)-), o-Cymene, Thymol, Gamma-Terpine, and Caryophyllene, with average concentration percentages of 18.03 ± 4.91, 12.77 ± 0.70, 7.90 ± 4.97, 6.61 ± 0.38, and 4.94 ± 0.09, respectively. With regard to the second chromatographic analysis, carvacrol showed an average percentage of 17.9 ± 1.55, whereas the determined average percentage of thymol was 7.7 ± 0.93. It is worth emphasizing, that for the second analysis, the oregano produced at the CGreenhouse exhibited the highest values of both terpenoids, while on the other hand, the CField site exhibited the lowest values of thymol percentage (1.7%) and Wild2 demonstrated the lowest value of carvacrol (13.8%) (Table 1b).

### 2.2. Antioxidant Activity of MEOE

The AoA of the MOEO was determined for four different concentrations: 0.10, 2.50, 5.00, and 10.00 µL mL^−1^, which are equivalent to 0.09, 2.30, 4.60, and 9.20 µg mL^−1^, respectively, at each sampling site. The results obtained show that the mean AoA for all sites was high (92.58%), with the concentration of 10.00 µL mL^−1^ standing out, having a mean AoA of 94.41% and a standard deviation (SD) of 0.28%. The lowest mean value was calculated for the 0.01 µL mL^−1^ concentration of MOEO with 90.19 ± 0.12%. In this same context, the Wild1 site obtained the highest average value of AoA with 95.61% at a concentration of 10.00 µL mL^−1^. Surprisingly, the lowest value was obtained for the same site (Wild1), showing an average AoA of 85.74% at a concentration of 0.10 µL mL^−1^ (Table 2).

### 2.3. Antibacterial Activity of MEOE

**Figure 1:**
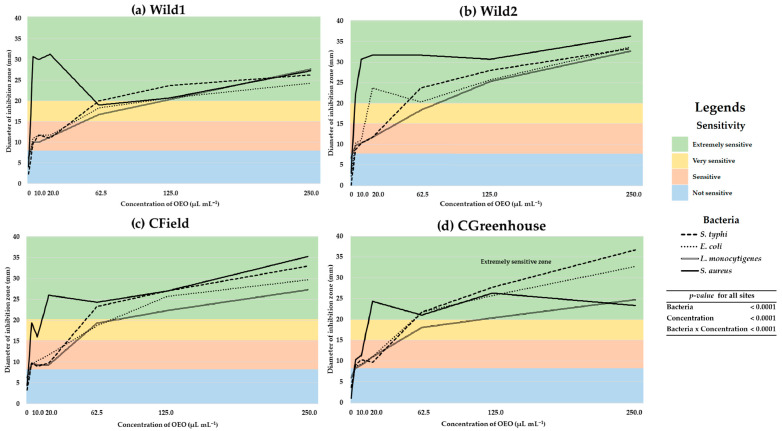
Antibacterial activity and sensitivity of Mexican oregano essential oil (*Lippia graveolens* Kunth) from plants occurring naturally in semiarid areas and cultivated in the field and greenhouse in northern Mexico.

The results regarding the AbA of the MOEO against the analyzed microorganisms were obtained by determining the sensitivity and the DIZ. The sensitivity to the different oil samples was classified based on the diameter of the inhibition halo as follows: not sensitive for diameters less than 8 mm; sensitive for diameters ranging from 9 to 14 mm; very sensitive for diameters between 15 and 19 mm; and extremely sensitive for diameters exceeding 20 mm [21]. To determine the AbA of the MOEO, nine different concentrations were analyzed at each sampling site for each of the four bacteria. In all the examined sites and bacteria, concentrations of 0.05, 0.10, and 1.00 µL mL^−1^ revealed DIZ values lower than 8.00 mm. However, upon analyzing the results for concentrations of 5.00 µL mL^−1^ and higher, the calculated DIZ was higher than 8 mm for all studied sites and bacteria (Figure 1).

The tendency in antimicrobial activity was very similar for the bacteria x site combination (Figure 1). Analyzing the obtained results, at a concentration of 1.00 µL mL^−1^, the only MOEO that reported a DIZ of 0.0 mm was the Wild2 site, and only for *Salmonella typhi* bacteria. However, surprisingly, the same site (Wild2) presented the highest DIZ (6.7 mm), but this time for the *Listeria monocytogenes* bacteria (Figure 1b). Based on the calculated DIZ of 5.00 µL mL^−1^, all the analyzed treatments exhibited significant AbA against both Gram-negative bacteria (*S. typhi* and *Escherichia coli*) and Gram-positive bacteria (*L. monocytogenes* and *Staphylococcus aureus*). Although no clear superiority was observed among the analyzed bacteria, the *S. aureus* bacteria showed the highest inhibition values within a range of MEOE concentrations between 20.00 and 62.50 µL mL^−1^. Considering the obtained results, at concentrations higher than the sensitivity, it was demonstrated that at an MOEO concentration of 10.00 µL mL^−1^, the Gram-positive bacteria *S. aureus* showed the highest DIZ values for all sites, achieving a maximum DIZ of 30.7 ± 1.5 mm for the MOEO obtained from the Wild2 site (Figure 1b). For this same concentration (10.00 µL mL^−1^), the lowest DIZ value was calculated for the combinations of the CGreenhouse site and the *L. monocytogenes* bacteria (Figure 1d) and the CField site and *S. typhi* bacteria (Figure 1c), both with a calculated mean DIZ value of 9.0 mm.

Considering the effect of a 20.00 µL mL^−1^ concentration of MOEO on the analyzed bacteria, it was determined that the combination of MOEO obtained from the Wild2 site and the *S. aureus* bacteria showed the highest mean value for the DIZ with 31.7 ± 0.6 mm (Figure 1b). Furthermore, at the same concentration, the lowest DIZ value was observed in the combination of the CField site and the *L. monocytogenes* bacteria with a mean DIZ of 9.3 ± 0.6 mm (Figure 1c). After analyzing the concentration of 62.50 µL mL^−1^, a homogenization process was observed in the DIZ values for all the analyzed sites, resulting in reduced differences between the highest and lowest DIZ values. Specifically, at this concentration, the combination of the CGreenhouse site with the *L. monocytogenes* bacteria showed the lowest DIZ value (Figure 1d), while the Wild2 site in conjunction with the *S. aureus* bacteria revealed the highest value (Figure 1b), with 18.0 ± 1.7 mm and 31.7 ± 4.5 mm, respectively. Considering the effect of the MOEO extracted from the CField site, the bacteria that showed the highest sensitivity to this oil were *S. aureus*, which reported the highest values of the DIZ at all the analyzed concentrations (Figure 1c). The concentrations of 125.00 and 250.00 µL mL^−1^ showed an increase in the DIZ. However, this growth was not proportional to the concentration increase. It was observed that the inhibition zone decreases with higher concentrations, indicating that the antibacterial potential of the MOEO at elevated concentrations is inversely proportional to the dilution of the oil (Figure 1).

## 3. Discussion

The results obtained in this study reveal that the MOEO (*L. graveolens*), derived from plants occurring naturally in semiarid areas and cultivated in the field and greenhouse in northern México, exhibits excellent AoA and AbA. However, in terms of both characteristics, the wild sites stood out in their performance. Based on these findings, our working hypothesis is supported.

### 3.1. Chemical Composition of MEOE

Normally, the yield and chemical composition of essential oils from plants of the same species show significant differences, mainly due to variations in environmental conditions, as reported for several species [46,47,48]. In this context, studying such variations in the EOs of species with economic and social importance, such as Mexican oregano (*L. graveolens*), is highly desirable. Chromatographic analysis of the EO of *L. graveolens* determined high percentages of thymol and carvacrol, suggesting that these are the major components in the oils from the four studied sites, which is consistent with findings reported in several studies [49,50]. Likewise, Cid-Pérez et al. [51] found in their chromatographic analysis that the OEO of *L. graveolens* presented high contents of thymol and carvacrol (28.31% and 17.06%), which are consistent only with the values calculated in this study for carvacrol (Table 1b). These findings indicate that despite belonging to the *L. graveolens* species, there is a notable difference in its chemical composition, possibly related to its phenology and production origin [52].

Studies conducted on OEO obtained from aerial parts of wild Mexican oregano reported 17.6% and 33.2% for thymol and carvacrol components, respectively [10]. These results differ from those found in the wild and cultivated oregano analyzed in this study but are similar to those observed in oregano obtained under controlled conditions (greenhouse) (Table 1). Similarly, other reports have indicated that cultivated oregano (*O. dictamnus*) in northern Mexico contains over 47% carvacrol and no thymol [33], indicating that wild oregano oil has significantly higher carvacrol levels compared with cultivated oregano in terms of thymol [16]. Sarrazin et al. [53] reported that carvacrol was the main component in the composition of the EO of *Lippia origanoides* produced during both the rainy and dry seasons, with 43.5% and 41.4%, respectively, followed by thymol, with 10.7% and 10.6%, indicating a low difference between the analyzed seasons. However, Walczak et al. [54] suggest that a high carvacrol content is desirable when aiming for specific biological activity. In another study, gas chromatography analysis of six OEOs of *O. vulgare* revealed percentages of 0.2% to 5.8% for thymol and 58.7% to 77.4% for carvacrol [55]. These findings show a lower calculated mean for thymol (7.7%) than in this study, whereas for carvacrol, the calculated values are lower than the mean reported in that study (17.9%). Additionally, a total percentage of 53.20% for the combined amount of carvacrol and thymol has been reported in *L. origanoides* leaves [56]; this amount is 107.8% higher than the one observed in the present study, where a mean of 25.6% was found for the sum of thymol and carvacrol percentages (Table 1).

### 3.2. Antioxidant Activity of MEOE

Considering that the scientific community has widely acknowledged the AoA of OEO, this research only used the ABTS method to compare the obtained oils. The results indicated that all analyzed oregano oils possess a high potential to neutralize free radicals (Table 2). The AoA of essential oils from different species known as oregano has been reported by several authors [3,16,17,20,22,47,55,56,57,58,59,60,61,62,63,64]. Although the results obtained in this study for the AoA of the EOE are comparable to those described in other regions of the world, they also turn out to be significantly higher than the published results (Table 3). This AoA has been related to the predominance of carvacrol and/or thymol in the EOE [62]; however, significant AoA has also been reported for other oxygenated compounds (monoterpenes) present in the EOE [64]. The differences observed in the reported and obtained results in this study are often attributed to the oregano species, the technique used for essential oil extraction, the concentrations used, the plant’s phenology, and the collection area of the plant material. However, the use of EOE as an antioxidant agent can enhance the preservation process of certain foods [65].

When comparing the AoA results obtained in this study with those reported by Flores-Martinez et al. [66], it is observed that they are similar. This similarity can be attributed to the AoA of MEOE (*L. graveolens*) being reported to be up to 90%, which is attributed to the quantities of thymol and carvacrol. These compounds synergistically enhance the antioxidant capacity, as explained by Paudel et al. [67], who identified that the EOE of the species *Origanum majorana* L. from sites in Nepal has a free radical inhibition capacity above 50%.

### 3.3. Antibacterial Activity of MEOE

After conducting the AbA tests on the different samples of MOEO, it was successfully demonstrated that the essential oil of *L. graveolens* significantly inhibits both Gram-positive and Gram-negative bacteria (Figure 1). In this regard, other studies that have examined the activity of OEO from different oregano species [3,4,22,29,46,48,50,51,68,69,70,71,72,73,74,75,76] have reported results consistent with those obtained in the present study. In this context, different research groups agree that the AbA of OEO is primarily associated with its hydrophobic phenolic compounds [77,78,79], based on the assumption that these compounds interact with the phospholipids present in the cell membrane. Zweifel et al. [80] attribute a higher resistance of Gram-negative bacteria to OEO to the complexity of the double cell membrane, contrasting it with the cell membrane structure of Gram-positive bacteria.

The obtained results evidenced an adequate AbA for all analyzed MEOEs and for all the bacteria considered (Figure 1). This activity may be related to the chemical composition of MEOE (Table 1). A mode of action of EOE compounds (especially thymol and carvacrol) has been reported, involving rapid depletion of intracellular ATP reserve. This results in a reduction in the proton motive force, enhancing membrane permeability, and ultimately leading to the leakage of ions from the cell, resulting in membrane damage and alterations in cellular osmotic pressure [22,81,82,83,84]. Different studies have demonstrated that carvacrol also inhibits the production of microbial toxins and the formation of biofilms, in addition to presenting anti-inflammatory effects [4,85,86]. A high carvacrol content in the OEO, combined with its water solubility, could contribute to a significant AbA effect [87]. In the same context, the only difference between carvacrol and thymol is the position of the hydroxyl group, which, along with the concentrations of other terpenes in OEO, would evidence a slightly different activity [4,88]. Existing research has shown that both carvacrol and thymol decrease cytoplasmic pH, damage the cell membrane, and increase membrane permeability in *S. aureus* [89].

This study demonstrated a significant AbA against the tested bacteria, with DIZ ranging from 0.0 to 6.7 mm for concentrations higher than 1.00 µL mL^−1^ and between 23.3 and 36.7 mm for the highest studied concentration (250.00 µL mL^−1^) (Figure 1). However, the MOEOs significantly differ in their activity against the bacteria considered in the study. This is evidenced based on the following: Analyzing the concentration of 1.00 µL mL^−1^, the combination of Wild2 EO and *L. monocytogenes* showed the highest DIZ at 6.7 mm; the combination of EO Wild1 and *S. aureus* exhibited the highest DIZ for the concentration of 5.00 µL mL^−1^ (30.7 mm) (Figure 1). Surprisingly, at the concentration of 10.00 µL mL^−1^, the combination of EO Wild2 and *S. aureus* reported the highest DIZ value at 30.70 mm. For the concentration of 20.00 µL mL^−1^, Wild1 and Wild2 EO, along with the *S. aureus* bacterium, showed the highest DIZ values of 31.3 mm and 31.7 mm, respectively. In general, the analyzed bacteria displayed sensitivity to the MOEO (Figure 1). This phenomenon has been reported by several research groups, for example, Ozkalp et al. [90] reported the effective antimicrobial activity (AmA) of the OEO in inhibiting the growth of *Micrococcus luteus* and *Bacillus cereus* at concentrations of 0.16 and 0.32 1.00 µL mL^−1^, respectively. According to De Falco et al. [91], the EOE, produced under different conditions, demonstrated effective AmA, primarily against Gram-positive pathogens, particularly *Bacillus cereus* and *Bacillus subtilis*. On the other hand, Khosravi et al. [92] reported that OEO exhibited a broad spectrum of antifungal activity, with an average DIZ of 27.1 mm against *Candida glabrata*. In another study conducted by Esen et al. [93], the OEO derived from wild oregano in the Marmara region of Turkey was effective in inhibiting the growth of both Gram-positive and Gram-negative bacteria, with values ranging from 0.63 to 5.0 µL mL^−1^. In the same way, De Martino et al. [71] reported that the EO of *O. vulgare* showed an adequate AbA against Gram-positive pathogens, with *S. epidermis* being the most affected (0.25 µL mL^−1^).

The differences observed among the cited results could likely be related to the extraction methods, composition of the OEO, varying sensitivity of different microorganisms to OEO, and specific climatic conditions during the production of each study. However, it appears evident that the AbA is primarily linked to a significant proportion of carvacrol and thymol, suggesting that its mechanism of action is similar to that of other phenolics [89,94]. This tendency is consistent with numerous studies investigating the effects of whole essential oils against organisms, particularly those that deteriorate food and foodborne pathogens [22,48,50,51,94]. Nevertheless, the use of OEO as an antioxidant agent may enhance the preservation process of specific foods [65] and can be effective against bacteria [95]. Additionally, OEO extracts can serve as synthetic chemical substitutes capable of inhibiting the growth of pathogenic organisms [96].

## 4. Materials and Methods

### 4.1. Study Location and Plant Material Identification

The sampling process included the collection of samples from four locations (Table 4), two of which were from communities of naturally occurring oregano plants (Wild1 and Wild2), one from plants cultivated in the field (CField), and one from plants cultivated in the greenhouse. All of these sites were located in the municipality of Rodeo, Durango, Mexico, at the geographical coordinates 25.1718° N and 104.5725° W, with an average altitude of 1345 m above sea level. The climate is semiarid, with an average annual temperature of 19.1 °C, a maximum of 25.5 °C, and a minimum of 12.1 °C. The average annual precipitation is 417.3 mm. The precipitation regime occurs mainly from June to September [97]. Oregano plants cultivated in the field received only two irrigations per year, one in November and another in April, during peak demand periods. On the other hand, oregano plants cultivated in the greenhouse were equipped with a drip irrigation system, receiving irrigation every two months with a water depth of 10 cm. The oregano seeds used for cultivation in the field and greenhouse were obtained from wild oregano populations in the region. It is worth noting that none of the studied sites received fertilization, insecticides, or herbicides. The collection was carried out on the same day for all four sites (21 October 2022). The entire aerial part of the plant was collected (stems, leaves, flowers, and fruits). Subsequently, it was dehydrated at room temperature and in the shade for four days, followed by the separation of the stems from the rest of the collected parts. The stems were discarded, and the remaining parts were weighed in triplicate, totaling 350.00 g, using an analytical balance from Denver Instrument Company (Arvada, CO, USA). Finally, they were labeled and stored in clean, sealed paper bags until their use.

Samples of mature oregano plants from the four studied sites were sent to and identified by the Botany and Systematics Laboratory of the Faculty of Biological Sciences at the Universidad Juarez del Estado de Durango, located in Gomez Palacio, Durango, Mexico. The results obtained from the identification showed that the plants from the four sampling sites correspond to Mexican oregano *(Lippia graveolens* Kunth).

### 4.2. Extraction of MOEO

The OEOs were extracted by hydrodistillation using a Clevenger apparatus [98]. The samples were immersed in 3000 mL of distilled water and subjected to a modified steam-stripping hydrodistillation method for two hours. After measuring the volume of the MOEO, the samples were dehydrated using anhydrous sodium sulfate. Subsequently, the density of the samples was determined using an analytical balance from Denver Instrument Company. Finally, they were stored in amber glass vials, wrapped in aluminum foil, and kept at 4 °C until use. All procedures were performed in triplicate.

### 4.3. Gas Chromatography Spectrometry Analysis of MOEO

To determine the main chemical components of each MOEO sample, an Agilent 7890B-59777A-MSD gas chromatography–mass spectrophotometry-coupled system was employed. The acquisition software used was MassHunter GC/MS Version Acquisition B.07.02.1938. Separation was carried out on an HP-SMS column (30.0 m × 0.25 mm × 0.25 µm internal diameter). Helium was used as the carrier gas at a flow rate of 1.0 mL min^−1^. The injector temperature used was 300 °C with an initial temperature (T_initial_) of 40 °C. The temperature programming for the separation included a T_initial_ of 40 °C for one minute, with a ramp rate of 15 °C min^−1^, reaching 300 °C with a split ratio of 200:1. The source temperature was 230 °C, the quadrupole temperature was 150 °C, and an ionization energy of 70 eV was used. The National Institute of Standards and Technology Mass Spectral Database, version 14.0, was employed for the identification of the reported components.

Considering that the concentrations of thymol and carvacrol in the OEO have received particular attention in the conducted study, additionally, and to reinforce the obtained results, a second chromatographic analysis was performed. This analysis was exclusively conducted to determine the concentrations of thymol and carvacrol in each of the obtained MOEO samples. For this purpose, a YL Instruments YL6500 gas chromatograph equipped with flame ionization detection (FID) and a MEGA-5 capillary column (60 m length × 0.25 mm in internal diameter, with a 0.15 μm film thickness) was used. The chromatograph employed a hydrogen mobile phase at a flow rate of 1 mL min^−1^. The injector and detector port temperatures were set at 250 °C and 300 °C, respectively, with a makeup of 20 mL min^−1^. The analysis started with an initial temperature of 40 °C and ramped at 10 °C, with a total run time of 16 min. Two microliters of the sample were injected for this determination.

### 4.4. Antioxidant Activity of MOEO

The AoA of the MOEOs was determined by UV-visible spectrophotometry (Thermo Scientific, Waltham, MA, USA) at 414 nm. The antioxidant assay tested was ABTS (2,2-azino-bis(3-ethylbenzothiazoline-6-sulfonate)) as described by Childs and Bardsley [99]. The MOEO samples were dissolved and agitated in 100 mL of dimethyl sulfoxide. Standard curves were prepared using Trolox as a potent antioxidant reagent. The samples were incubated for 10 min at 37 °C and cooled on ice. The absorbance of Trolox at different concentrations was measured at 414 nm. All results were then calculated and expressed as mg of Trolox equivalents per mL of the MOEO sample.

### 4.5. Antibacterial Activity of MOEO

#### 4.5.1. Bacterial Strains

The microbiological material used consisted of four pathogenic bacterial strains responsible for certain serious infectious diseases. These bacteria were *Salmonella typhi* ATCC 14028, *Escherichia coli* ATCC 25922, *Listeria monocytogenes* ATCC 7644, and *Staphylococcus aureus* ATCC 6538. These strains were cultivated in brain–heart infusion broth and sterilized by autoclaving at their respective optimal growth temperatures.

#### 4.5.2. Agar Diffusion Method

The AbA of the MOEO was evaluated using the agar diffusion method according to the recommendations of NCCLS [100]. Bacterial strains were prepared in appropriate culture media and according to international standards. The microwell technique, described by Rossi et al. [101], was used, for which a serial dilution of 10:3 was performed, with 1 mL of the strain in 9 mL of phosphate-buffered solution. Afterward, 8.5 mL of the dilution was added to 250 mL of Müller–Hinton agar. The mixture was gently stirred to homogenize it before being poured into pre-sterilized Petri dishes and autoclaved at 125 °C for 15 min. Subsequently, it was incubated at 36 °C for 18 h.

#### 4.5.3. Determination of Diameter of Inhibition Zone (DIZ) and Sensitivity

The sensitivity of the MOEO for each bacterium was evaluated according to Moreira et al. [21]. The MOEO was diluted using dimethyl sulfoxide (DMSO) to obtain nine different concentrations (0.05, 0.10, 1.00, 5.00, 10.00, 20.00, 62.50, 125.00, and 250.00 µL mL^−1^). The microwells were prepared using a sterile 100 µL tip, following the method of Wiegan et al. [102]. The study was performed in triplicate, using DMSO as the negative control and Trimethoprim-Sulfamethoxazole as the positive control [103]. Microbial suspensions were prepared according to the following standards: 0.5 McFarland, which is equivalent to 10^8^ colony-forming unit mL^−1^ [104]. Subsequently, 0.1 mL of the inoculum was placed on the agar using a sterile swab. Then, the MOEO concentrations were directly placed on the agar surface. The Petri dishes were incubated in a stove at 37 °C for 24 h. The DIZ values of the four MOEOs against the four bacteria used were determined using the recommended method described at the beginning of the paragraph. After incubating the Petri dishes, the DIZ value was measured in millimeters using Vernier calipers. All experiments were repeated in triplicate.

### 4.6. Statistical Analysis

The data were expressed as mean ± standard deviation (SD). A univariate linear model (ANOVA) was performed using the statistical software SPSS^®^ version 2020 [105]. A 95% confidence interval (*p* < 0.05) was considered statistically significant.

## 5. Conclusions

Currently, several industries are seeking new, natural, and safe agents. The determination of the AoA and AbA of OEO from *L. graveolens* produced under different conditions in northern Mexico has revealed its potential as an excellent natural agent. The results suggest that *L. graveolens* OEO contains compounds with both antimicrobial and antioxidant properties, positioning it as a strong candidate for use in food preservation and/or shelf life extension. Moreover, the significant potential of the *L. graveolens* oregano species is evident for applications such as tea preparation, food additives, and traditional remedies for treating infectious diseases. Similarly, there is a need for further investigation into the AoA and AbA of MOEO, particularly by determining the minimum bactericidal and bacteriostatic concentration. However, future research should aim to explore the potential effects of climatic conditions and soil properties at the sampling sites on the biosynthesis of terpenoids, especially thymol and carvacrol. These insights contribute to the utilization of *L. graveolens* OEO as a functional food and pharmacological ingredient to enhance health. This will contribute to mitigating the effects of climate change and improving food security, in line with the goals of the sustainable development agenda for 2030.

## Figures and Tables

**Table 1:** (**a**) Percentages in the sample of the main chemical components in Mexican oregano essential oil (*Lippia graveolens* Kunth) from plants occurring naturally in semiarid areas and cultivated in the field and greenhouse in northern Mexico, analyzed with gas chromatography–mass spectrophotometry. (**b**) Relative composition of thymol and carvacrol in Mexican oregano essential oil (*Lippia graveolens* Kunth) from plants occurring naturally in semiarid areas and cultivated in the field and greenhouse in northern Mexico, analyzed with gas chromatography.

(**a**)						
**Num.**	**T_R_ (min)**	**Compound Name**	**Percentage in the Sample (Relative Areas)**			
			**Wild1**	**Wild2**	**Cfield**	**CGreenhouse**
1	4.39	Bicyclo[3.1.0]hexan-3-one, 4-methyl-1-(1-methylethyl)-	0.31	0.32	0.39	0.41
2	4.70	Tricyclo[2.2.1.0(2,6)]heptane, 1,3,3-trimethyl-	0.43	0.47	0.42	0.42
3	5.08	Beta-myrcene	4.40	4.50	4.50	4.50
4	5.37	(+)-4-Carene	1.87	1.89	2.01	1.90
5	5.46	o-Cymene	13.42	13.24	11.90	12.50
6	5.51	d-Limonene	1.50	1.60	1.60	1.90
7	5.54	Eucalyptol	1.66	1.65	2.01	1.50
8	5.83	Gamma-Terpine	6.23	7.10	6.70	6.41
9	5.91	Bicyclo[3.1.0]hexan-2-ol, 2-methyl-5-(1-methylethyl)-, (1α,2β,5α)-	0.20	0.21	0.31	0.30
10	6.14	cyclohexane 1-methyl-4-(1-methylethylidene)-	0.26	0.31	0.28	0.27
11	6.24	Linalool	0.95	0.98	1.02	1.02
12	7.06	3-cyclohexen-1-ol 4-methyl-1-(1-methylethyl)- (r)-	1.79	1.80	2.01	1.84
13	7.22	Alpha-terpineol	0.27	0.30	0.29	0.28
14	7.59	Benzene 2-methoxy-4-methyl-1-(1-methylethyl)-	0.16	0.17	0.17	0.15
15	8.16	Thymol	8.90	6.90	1.90	13.90
16	8.23	Carvacrol	16.60	14.10	16.20	25.20
17	8.74	Eugenol	0.31	0.42	0.33	0.32
18	9.38	Caryophyllene	4.83	5.01	5.01	4.90
19	9.46	*cis*-alpha-bergamotene	1.14	1.10	2.00	1.20
20	9.66	Humulene	2.32	2.33	3.14	3.01
21	9.79	Phenol, 3-(1,1-dimethylethyl)-4-methoxy	0.41	0.39	0.51	0.42
22	10.06	β-Bisabolene	0.20	0.30	0.22	0.21
23	10.72	Caryophyllene oxide	0.76	0.79	0.79	0.78
24	10.92	(1*R*,3*E*,7*E*,11*R*)-1,5,5,8-Tetramethyl-12-oxabicyclo[9.1.0]dodeca-3,7-diene	0.28	0.26	0.30	0.28
(**b**)						
**Site**			**Thymol (%)**		**Carvacrol (%)**	
Wild1			8.4 ± 1.02 ^ab^		16.4 ± 1.30 ^ab^	
Wild2			6.6 ± 0.99 ^b^		13.8 ± 1.83 ^ab^	
CField			1.7 ± 0.04 ^c^		15.7 ± 1.06 ^ab^	
CGreenhouse			14.1 ± 1.680 ^a^		25.7 ± 2.02 ^a^	

^a,b,c^ Different lowercase letters represent significant differences (*p* < 0.05) between sites.

**Table 2:** Antioxidant activity (%±SD) of Mexican oregano essential oil (*Lippia graveolens* Kunth) from plants occurring naturally in semiarid areas and cultivated in the field and greenhouse in northern Mexico.

Site/ Concentration (µL mL^−1^)	ABTS Antioxidant Activity (%)			
	0.01	2.50	5.00	10.00
Wild1	85.74 ± 0.21 ^a^	90.69 ± 0.18 ^b^	93.44 ± 0.54 ^b^	95.61 ± 0.53 ^c^
Wild2	90.80 ± 0.01 ^a^	93.28 ± 0.11 ^a^	93.28 ± 0.11 ^b^	95.27 ± 0.10 ^b^
CField	91.47 ± 0.05 ^b^	90.42 ± 0.13 ^b^	93.40 ± 0.20 ^b^	92.27 ± 0.20 ^b^
CGreenhouse	92.76 ± 0.19 ^b^	93.05 ± 0.01 ^b^	93.33 ± 0.11 ^b^	94.50 ± 0.28 ^b^

^a,b,c^ Different lowercase letters represent significant differences (*p* < 0.05) between sites x concentration.

**Table 3:** Antioxidant activity average (%, AoA) generated by the Mexican oregano essential oil (*Lippia graveolens* Kunth) from plants occurring naturally in semiarid areas and cultivated in the field and greenhouse in northern Mexico, compared to other reported studies on oregano species.

Specie	Production System	Assay Type to Calculate the AoA ^1^	% AoA	Region, Country
*Lippia graveolens*	PNO ^2^	ABTS ^5^	93.4%	Durango, Mexico
	CField ^3^		91.9%	
	CGH ^4^		92.3%	
*Poliomintha longiflora*	CField	ORAC ^6^	34.7%	Nuevo Leon, Mexico [16]
	PNO		55.6%	
*Origanum vulgare*	CField	DPPH ^7^	61.7–62.0%	Hubei, China [17]
		ABTS	91.5–95.9%	
Oregano (Scientific name NS ^8^)	NS	DPPH	79.0–99.8%	Collection site NS [20]
*Origanum vulgare* spp. *hirtum*	CField	ABTS	8.5%	Warszawa, Poland [22]
*Origanum vulgare* spp. *vulgare*			8.6%	
*Origanum vulgare*	NS	DPPH	12.9–47.3%	Uttarakhand, India [46]
*Origanum vulgare*	CField	ABTS	35.0–62.5%	Murcia, Spain [55]
*Origanum vulgare*	PNO	ORAC	52%	Alentejo, Portugal [57]
*Poliomintha longiflora*	PNO	ORAC	92.2%	USA [58]
*Origanum vulgare* spp. *vulgare*			64.7%	
Oregano (Scientific name NS)	NS	ORAC	50%	USA [59]
*Origanum vulgare*	CField	ORAC	39.8–84.8%	Gatersleben, Germany [60]

^1^ AoA = antioxidant activity; ^2^ PNO = plants naturally occurring; ^3^ CField = cultivated in the field; ^4^ CGH = cultivated in the greenhouse; ^5^ ABTS = 2,2-azino-bis (3-ethylbenzothiazoline-6-sulfonate); ^6^ ORAC = oxygen radical absorbance capacity; ^7^ DPPH = 2,2-diphenylpicrylhydrazyl hydrate; ^8^ NS = Not specified.

**Table 4:** Geographical characteristics and habitats of sampling sites.

Site	Latitude (N)	Longitude (W)	Altitude (m)	Soil Type	Habitat
Wild1	25.0233°	104.4758°	1471	Phaeozem	Microphyll scrub, semiarid
Wild2	25.1786°	104.5686°	1446	Chernozem	Microphyll scrub, semiarid
CField	25.2386°	104.5675°	1338	Fluvisol	Irrigation agriculture
CGreenhouse	25.1650°	104.5558°	1342	Chernozem	Controlled conditions

## Data Availability

Not applicable.

## References

[1] Yuan Y., Sun J., Song Y., Raka R.N., Xiang J., Wu H., Xiao J., Jin J., Hui X. (2023). Antibacterial Activity of Oregano Essential Oils against *Streptococcus mutans* In Vitro and Analysis of Active Components. BMC Complement. Med. Ther..

[2] Morshedloo M.R., Salami S.A., Nazeri V., Maggi F., Craker L. (2018). Essential Oil Profile of Oregano (*Origanum vulgare* L.) Populations Grown under Similar Soil and Climate Conditions. Ind. Crops Prod..

[3] Castilho P.C., Savluchinske-Feio S., Weinhold T.S., Gouveia S.C. (2012). Evaluation of the Antimicrobial and Antioxidant Activities of Essential Oils, Extracts and Their Main Components from Oregano from Madeira Island, Portugal. Food Control.

[4] Man A., Santacroce L., Iacob R., Mare A., Man L. (2019). Antimicrobial Activity of Six Essential Oils Against a Group of Human Pathogens: A Comparative Study. Pathogens.

[5] Bautista-Hernandez I., Aguilar C.N., Martinez-Avila G.C.G., Torres-Leon C., Ilina A., Flores-Gallegos A.C., Kumar Verma D., Chavez-Gonzalez M.L. (2021). Mexican Oregano (*Lippia graveolens* Kunth) as Source of Bioactive Compounds: A Review. Molecules.

[6] Comision Nacional Forestal—National Forestry Commission (CONAFOR) Catalogo de Recursos Forestales Maderables y No Maderables: Arido, Tropical y Templado. http://www.conafor.gob.mx/biblioteca/Catalogo_de_recursos_forestales_M_y_N.pdf.

[7] Lawrence B.M. (1984). The Botanical and Chemical Aspects of Oregano. Perfum. Flavorist.

[8] Russo M., Galletti G.C., Bocchini P., Carnacini A. (1998). Essential Oil Chemical Composition of Wild Populations of Italian Oregano Spice (*Origanum vulgare* ssp. *hirtum* (Link) Ietswaart): A Preliminary Evaluation of Their use in Chemotaxonomy by Cluster Analysis. 1. Inflorescences. J. Agric. Food Chem..

[9] Granata G., Stracquadanio S., Leonardi M., Napoli E., Malandrino G., Cafiso V., Stefani S., Geraci C. (2021). Oregano and Thyme Essential Oils Encapsulated in Chitosan Nanoparticles as Effective Antimicrobial Agents Against Foodborne Pathogens. Molecules.

[10] Cid-Perez T.S., Torres-Munoz J.V., Nevarez-Moorillon G.V., Palou E., Lopez-Malo A. (2016). Chemical Characterization and Antifungal Activity of *Poliomintha longiflora* Mexican Oregano. J. Essent. Oil Res..

[11] Hao Y., Li J., Zhang W., Sun M., Li H., Xia F., Cui H., Bai H., Shi L. (2021). Analysis of the Chemical Profiles and Anti-*S. aureus* Activities of Essential Oils Extracted from Different Parts of Three Oregano Cultivars. Foods.

[12] Chacon-Vargas K.F., Sanchez-Torres L.E., Chavez-Gonzalez M.L., Adame-Gallegos J.R., Nevarez-Moorillon G.V. (2022). Mexican Oregano (*Lippia berlandieri* Schauer and *Poliomintha longiflora* Gray) Essential Oils Induce Cell Death by Apoptosis in *Leishmania* (*Leishmania*) *mexicana* Promastigotes. Molecules.

[13] Ortega-Lozano A.J., Hernandez-Cruz E.Y., Gomez-Sierra T., Pedraza-Chaverri J. (2023). Antimicrobial Activity of Spices Popularly Used in Mexico Against Urinary Tract Infections. Antibiotics.

[14] Leyva-Lopez N., Gutierrez-Grijalva E.P., Vazquez-Olivo G., Heredia J.B. (2017). Essential Oils of Oregano: Biological Activity Beyond their Antimicrobial Properties. Molecules.

[15] Cui H., Zhang C., Li C., Lin L. (2019). Antibacterial Mechanism of Oregano Essential Oil. Ind. Crops Prod..

[16] Mora-Zuñiga A.E., Trevino-Garza M.Z., Amaya Guerra C.A., Galindo Rodriguez S.A., Castillo S., Martinez-Rojas E., Rodriguez-Rodriguez J., Baez-Gonzalez J.G. (2022). Comparison of Chemical Composition, Physicochemical Parameters, and Antioxidant and Antibacterial Activity of the Essential Oil of Cultivated and Wild Mexican Oregano *Poliomintha longiflora* Gray. Plants.

[17] Shen Y., Zhou J., Yang C., Chen Y., Yang Y., Zhou C., Wang L., Xia G., Yu X., Yang H. (2022). Preparation and Characterization of Oregano Essential Oil-Loaded *Dioscorea zingiberensis* Starch Film with Antioxidant and Antibacterial Activity and its Application in Chicken Preservation. Int. J. Biol. Macromol..

[18] Luo K., Zhao P., He Y., Kang S., Shen C., Wang S., Guo M., Wang L., Shi C. (2022). Antibacterial Effect of Oregano Essential Oil against *Vibrio vulnificus* and Its Mechanism. Foods.

[19] Li B., Zheng K., Lu J., Zeng D., Xiang Q., Ma Y. (2022). Antibacterial Characteristics of Oregano Essential Oil and its Mechanisms Against *Escherichia coli* O157: H7. J. Food Meas. Charact..

[20] Wu M., Zhou Z., Yang J., Zhang M., Cai F., Lu P. (2021). ZnO Nanoparticles Stabilized Oregano Essential Oil Pickering Emulsion for Functional Cellulose Nanofibrils Packaging Films with Antimicrobial and Antioxidant Activity. Int. J. Biol. Macromol..

[21] Moreira M.R., Ponce A.G., Del Valle C.E., Roura S.I. (2005). Inhibitory Parameters of Essential Oils to Reduce a Foodborne Pathogen. LWT-Food Sci. Technol..

[22] Kosakowska O., Węglarz Z., Pióro-Jabrucka E., Przybył J.L., Kraśniewska K., Gniewosz M., Bączek K. (2021). Antioxidant and Antibacterial Activity of Essential Oils and Hydroethanolic Extracts of Greek Oregano (*O. vulgare* L. subsp. *hirtum* (Link) Ietswaart) and Common Oregano (*O. vulgare* L. subsp. *vulgare*). Molecules.

[23] Baycheva S.K., Dobreva K.Z. (2021). Chemical Composition of Bulgarian White Oregano (*Origanum heracleoticum* L.) Essential Oils. IOP Conf. Ser. Mater. Sci. Eng..

[24] Lan W., Zhao X., Chen M., Xie J. (2022). Antimicrobial Activity and Mechanism of Oregano Essential Oil against *Shewanella putrefaciens*. J. Food Saf..

[25] Bhat V., Sharma S.M., Shetty V., Shastry C.S., Rao C.V., Shenoy S., Saha S., Balaji S. (2018). Characterization of Herbal Antifungal Agent, *Origanum vulgare* against Oral *Candida* spp. Isolated from Patients with Candida-Associated Denture Stomatitis: An In Vitro Study. Contemp. Clin. Dent..

[26] Salgado-Nava A.A., Hernandez-Nava R., Lopez-Malo A., Jimenez-Munguia M.T. (2020). Antimicrobial Activity of Encapsulated Mexican Oregano (*Lippia berlandieri* Schauer) Essential Oil Applied on Bagels. Front. Sustain. Food Syst..

[27] Meenu M., Padhan B., Patel M., Patel R., Xu B. (2022). Antibacterial Activity of Essential Oils from Different Parts of Plants against *Salmonella* and *Listeria* spp. Food Chem..

[28] Marin-Tinoco R.I., Camacho-Luis A., Silva-Marrufo O., Diaz-Diaz M., Ortega-Ramirez A.T. (2021). Inhibition of *Candida albicans* by Oregano (*Lippia* spp.) Essential Oil from Municipality of Rodeo, Durango, Mexico. J. Microbiol. Health Educ..

[29] Jan S., Rashid M., Abd_Allah E.F., Ahmad P. (2020). Biological Efficacy of Essential Oils and Plant Extracts of Cultivated and Wild Ecotypes of *Origanum vulgare* L. BioMed. Res. Int..

[30] Goudjil M.B., Zighmi S., Hamada D., Mahcene Z., Bencheikh S.E., Ladjel S. (2020). Biological Activities of Essential Oils Extracted From *Thymus capitatus* (Lamiaceae). S. Afr. J. Bot..

[31] Shanaida M., Golembiovska O. (2018). Identification and Component Analysis of Triterpenoids in *Monarda fistulosa* L. and *Ocimum americanum* L. (Lamiaceae) Aerial Parts. Pharm. Sci..

[32] Ventura S.P.M., Silva F.A., Quental M.V., Mondal D., Freire M.G., Coutinho J.A.P. (2017). Ionic-LiquidMediated Extraction and Separation Processes for Bioactive Compounds: Past, Present and Future Trends. Chem. Rev..

[33] Olivas N.A., Bejarano C.V., Soto G.A., Ortega M.Z., Salas F.S., Chávez E.S., Ochoa L.H. (2020). Bioactive Compounds and Antioxidant Activity of Essential Oils of *Origanum dictamnus* From Mexico. AIMS Agric. Food.

[34] Cortes-Chitala M.d.C., Flores-Martinez H., Orozco-Avila I., Leon-Campos C., Suarez-Jacobo A., Estarron-Espinosa M., Lopez-Muraira I. (2021). Identification and Quantification of Phenolic Compounds from Mexican Oregano (*Lippia graveolens* HBK) Hydroethanolic Extracts and Evaluation of Its Antioxidant Capacity. Molecules.

[35] Shahin S.M., Jaleel A., Alyafei M.A.M. (2021). Yield and In Vitro Antioxidant Potential of Essential Oil from *Aerva javanica* (Burm. f.) Juss. ex Schul. Flower with Special Emphasis on Seasonal Changes. Plants.

[36] Marin-Tinoco R.I., Silva-Marrufo O., Gonzales-Güereca M. (2019). Physical characterization-chemistry of essential oil of oregano in 6 communities of the Municipality of Rodeo, Dgo. J. Urban Rural Reg. Econ..

[37] Manso S., Becerril R., Nerin C., Gomez-Lus R. (2015). Influence of pH and Temperature Variations on Vapor Phase Action of an Antifungal Food Packaging against Five Mold Strains. Food Control.

[38] Muriel-Galet V., Cran M.J., Bigger S.W., Hernandez-Munoz P., Gavara R. (2015). Antioxidant and Antimicrobial Properties of Ethylene Vinyl Alcohol Copolymer Films Based on the Release of Oregano Essential Oil and Green Tea Extract Components. J. Food Eng..

[39] Elshafie H.S., Armentano M.F., Carmosino M., Bufo S.A., De Feo V., Camele I. (2017). Cytotoxic Activity of *Origanum vulgare* L. on Hepatocellular Carcinoma Cell Line HepG2 and Evaluation of its Biological Activity. Molecules.

[40] Llana-Ruiz-Cabello M., Maisanaba S., Puerto M., Pichardo S., Jos A., Moyano R., Camean A.M. (2017). A Subchronic 90-day Oral Toxicity Study of *Origanum vulgare* Essential Oil in Rats. Food Chem. Toxicol..

[41] Efrati R., Natan M., Pelah A., Haberer A., Banin E., Dotan A., Ophir A. (2014). The Combined Effect of Additives and Processing on the Thermal Stability and Controlled Release of Essential Oils in Antimicrobial Films. J. Appl. Polym. Sci..

[42] Navarrete-Molina C., Meza-Herrera C.A., Ramirez-Flores J.J., Herrera-Machuca M.A., Lopez-Villalobos N., Lopez-Santiago M.A., Veliz-Deras F.G. (2019). Economic Evaluation of the Environmental Impact of a Dairy Cattle Intensive Production Cluster under Arid Lands Conditions. Animal.

[43] Navarrete-Molina C., Meza-Herrera C.A., Herrera-Machuca M.A., Lopez-Villalobos N., Lopez-Santos A., Veliz-Deras F.G. (2019). To Beef or Not To Beef: Unveiling the Economic Environmental Impact Generated by the Intensive Beef Cattle Industry in an Arid Region. J. Clean. Prod..

[44] Navarrete-Molina C., Meza-Herrera C.A., Herrera-Machuca M.A., Macias-Cruz U., Veliz-Deras F.G. (2020). Not All Ruminants Were Created Equal: Environmental and Socio-Economic Sustainability of Goats Under a Marginal-Extensive Production System. J. Clean. Prod..

[45] Rios-Flores J.L., Rios-Arredondo B.E., Cantu-Brito J.E., Rios-Arredondo H.E., Armendariz-Erives S., Chavez-Rivero J.A., Navarrete-Molina C., Castro-Franco R. (2018). Analisis de la Eficiencia Fisica, Economica y Social del Agua en Esparrago (*Asparagus officinalis* L.) y Uva (*Vitis vinifera*) de Mesa del DR-037 Altar-Pitiquito-Caborca, Sonora, Mexico 2014. Rev. Fac. Cienc. Agrar..

[46] Bhatt S., Tewari G., Pande C., Prakash O., Tripathi S. (2019). Aroma Profile and Antioxidant Potential of *Origanum vulgare* L.: Impact of Drying. J. Essent. Oil Bear Plants.

[47] Fikry S., Khalil N., Salama O. (2019). Chemical Profiling, Biostatic and Biocidal Dynamics of *Origanum vulgare* L. Essential Oil. AMB Express.

[48] Tanasescu S., Nitu R., Dahma G., Pilut C., Diaconu M., Neagoe O., Muntean D., Horhat I.D., Dragomir A., Lighezan D. (2019). Chemical Composition and Antimicrobial Activity of Essential Oil of Romanian *Origanum vulgare*. Rev. Chim..

[49] Quintanilla-Licea R., Mata-Cardenas B.D., Vargas-Villarreal J., Bazaldua-Rodriguez A.F., Angeles-Hernandez I.K., Garza-Gonzalez J.N., Hernandez-Garcia M.E. (2014). Antiprotozoal Activity Against Entamoeba Histolytica of Plants Used in Northeast Mexican Traditional Medicine Bioactive Compounds from *Lippia graveolens* and *Ruta chalepensis*. Molecules.

[50] Oniga I., Puscas C., Silaghi-Dumitrescu R., Olah N.K., Sevastre B., Maricá R., Marco I., Sevastre-Berghian A.C., Benedec D., Pop C.E. (2018). *Origanum vulgare* ssp. *vulgare*: Chemical Composition and Biological Studies. Molecules.

[51] Cid-Perez T.S., Avila-Sosa R., Ochoa-Velasco C.E., Rivera-Chavira B.E., Nevarez-Moorillon G.V. (2019). Antioxidant and Antimicrobial Activity of Mexican Oregano (*Poliomintha longiflora*) Essential Oil, Hydrosol and Extracts from Waste Solid Residues. Plants.

[52] Tellez-Monzon L.A., Nolazco-Cama D.M. (2017). Estudio de la Composicion Quimica del Aceite Esencial de Oregano (*Origanum vulgare* spp.) de Tacna. Ing. Ind..

[53] Sarrazin S.L.F., Da Silva L.A., De Assunção A.P.F., Oliveira R.B., Calao V.Y.P., Da Silva R., Stashenko E.E., Maia J.G.S., Mourão R.H.V. (2015). Antimicrobial and Seasonal Evaluation of the Carvacrol-Chemotype Oil from *Lippia origanoides* Kunth. Molecules.

[54] Walczak M., Michalska-Sionkowska M., Olkiewicz D., Tarnawska P., Warzynska O. (2021). Potential of Carvacrol and Thymol in Reducing Biofilm Formation on Technical Surfaces. Molecules.

[55] Carrasco A., Perez E., Cutillas A.-B., Martinez-Gutierrez R., Tomas V., Tudela J. (2016). *Origanum vulgare* and *Thymbra capitata* Essential Oils from Spain: Determination of Aromatic Profile and Bioactivities. Nat. Prod. Commun..

[56] Muñoz-Acevedo A., Castañeda M.L., Blanco K.M., Cardenas C.Y., Reyes J.A., Kouznetsov V.V., Stashenko E.E. (2007). Composicion y Capacidad Antioxidante de Especies Aromaticas y Medicinales con Alto Contenido de Timol y Carvacrol. Sci. Technol..

[57] da Costa S.B., Duarte C., Bourbon A.I., Pinheiro A.C., Serra A.T., Martins M.M., Januário M.I.N., Vicente A.A., Delgadillo I., Duarte C. (2012). Effect of the Matrix System in the Delivery and In Vitro Bioactivity of Microencapsulated Oregano Essential Oil. J. Food Eng..

[58] Zheng W., Wang S.Y. (2001). Antioxidant Activity and Phenolic Compounds in Selected Herbs. J. Agric. Food Chem..

[59] Bhagwat S., Haytowitz D.B., Holden J.M. (2010). USDA Database for the Oxygen Radical Capacity (ORAC) of Selected Foods, Release 2. USDA National Nutrient Database for Standard Reference. http://www.ars.usda.gov/ARSUserFiles/80400525/Articles/AICR07_ORAC.pdf.

[60] Yan F., Azizi A., Janke S., Schwarz M., Zeller S., Honermeier B. (2016). Antioxidant Capacity Variation in the Oregano (*Origanum vulgare* L.) Collection of the German National Genebank. Ind. Crops Prod..

[61] Kulisic T., Radonic A., Katalinic V., Milos M. (2004). Use of Different Methods for Testing Antioxidative Activity of Oregano Essential Oil. Food Chem..

[62] Milos M., Mastelic J., Jerkovic I. (2000). Chemical Composition and Antioxidant Effect of Glycosidically Bound Volatile Compounds from Oregano (*Origanum vulgare* L. ssp. *hirtum*). Food Chem..

[63] Tuttolomondo T., Salvatore L.B., Licata M., Virga G., Leto C., Saija A., Trombetta D., Tomaino A., Speciale A., Napoli E.M. (2013). Biomolecular Characterization of Wild Sicilian Oregano: Phytochemical Screening of Essential Oils and Extracts, and Evaluation of their Antioxidant Activities. Chem. Biodivers..

[64] Ruberto G., Baratta M.T. (2000). Antioxidant Activity of Selected Essential Oil Components in Two Lipid Model Systems. Food Chem..

[65] Abdel-Khalek A.M. (2013). Supplemental Antioxidants in Rabbit Nutrition: A Review. Livest. Sci..

[66] Flores-Martinez H., Leon-Campos C., Estarron-Espinosa M., Orozco-Avila I. (2016). Process Optimization for the Extraction of Antioxidants from Mexican Oregano (*Lippia graveolens* Hbk) by the Response Surface Methodology (RSM) Approach. Rev. Mex. Ing. Quim..

[67] Paudel P.N., Satyal P., Satyal R., Setzer W.N., Gyawali R. (2022). Chemical Composition, Enantiomeric Distribution, Antimicrobial and Antioxidant Activities of *Origanum majorana* L. Essential Oil from Nepal. Molecules.

[68] Kalemba D., Kunicka A. (2003). Antibacterial and Antifungal Properties of Essential Oils. Curr. Med. Chem..

[69] Bagamboula C.F., Uyttendaele M., Debevere J. (2003). Antimicrobial Effect of Spices and Herbs on *Shigella sonnei* and *Shigella flexneri*. J. Food Prot..

[70] Bozin B., Mimica-Dukic N., Simin N., Anackov G. (2006). Characterization of the Volatile Composition of Essential Oils of Some Lamiaceae Spices and the Antimicrobial and Antioxidant Activities of the Entire Oils. J. Agric. Food Chem..

[71] De Martino L., De Feo V., Formisano C., Mignola E., Senatore F. (2009). Chemical Composition and Antimicrobial Activity of the Essential Oils from Three Chemotypes of *Origanum vulgare* L. ssp. *hirtum* (Link) Ietswaart Growing Wild in Campania (Southern Italy). Molecules.

[72] Hashemi S.M.B., Nikmaram N., Esteghlal S., Khaneghah A.M., Niakousari M., Barba F.J., Roohinejad S., Koubaa M. (2017). Efficiency of Ohmic Assisted Hydrodistillation for the Extraction of Essential Oil from Oregano (*Origanum vulgare* subsp. *viride*) Spices. Innov. Food Sci. Emerg. Technol..

[73] Benedec D., Hanganu D., Oniga I., Tiperciuc B., Olah N., Raita O., Bischin C., Silaghi R., Vlase L. (2015). Assessment of Rosmarinic Acid Content in Six Lamiaceae Species Extracts and their Antioxidant and Antimicrobial Potential. Pak. J. Pharm. Sci..

[74] Benedec D., Oniga I., Cuibus F., Sevastre B., Stiufiuc G., Duma M., Hanganu D., Iacovita C., Stiufiuc R., Lucaciu C.M. (2018). *Origanum vulgare* Mediated Green Synthesis of Biocompatible Gold Nanoparticles Simultaneously Possessing Plasmonic, Antioxidant and Antimicrobial Properties. Int. J. Nanomed..

[75] Bunghez F., Rotar M.A., Pop R.M., Romanciuc F., Csernatoni F., Fetea F., Diaconeasa Z., Socaciu C. (2015). Comparative Phenolic Fingerprint and LC-ESI+QTOF-MS Composition of Oregano and Rosemary Hydrophilic Extracts in Relation to their Antibacterial Effect. Bull. UASVM Food Sci. Technol..

[76] Yotova I., Ignatova-Ivanova T. (2015). In Vitro Study of Antifungal Activity of Oregano (*Origanum vulgare*). Int. J. Curr. Microbiol. Appl. Sci..

[77] Ozcan M., Erkmen O. (2001). Antimicrobial Activity of the Essential Oils of Turkish Plant Spice. Eur. Food Res. Technol..

[78] Sökmen M., Serkedjieva J., Daferera D., Gulluce M., Polissiou M., Tepe B., Akpulat A., Sahin F., Sokmen A. (2004). In Vitro Antioxidant, Antimicrobial, and Antiviral Activities of the Essential Oil and Various Extracts from Herbal Parts and Callus Cultures of *Origanum acutidens*. J. Agric. Food Chem..

[79] Paredes-Aguilar M.D.L.C., Gastelum-Franco M.G., Silva-Vazquez R., Nevarez-Moorillon G.V. (2007). Efecto Antimicrobiano del Oregano Mexicano (*Lippia berlandieri* Schauer) y de su Aceite Esencial Sobre Cinco Especies del Genero Vibrio. Rev. Fitotec. Mex..

[80] Zweifel C., Stephan R. (2012). Spices and herbs as source of Salmonella-related foodborne diseases. Food Res. Int..

[81] Franz C., Baser K.H.C., Windisch W. (2009). Essential Oils and Aromatic Plants in Animal Feeding—A European Perspective. A Review. Flavour Fragr. J..

[82] Veldhuizen E.J.A., Tjeersma van Bokhoven J.L.M., Zweijtzer C., Burt S.A., Haagsman H.P. (2006). Structural Requirements for the Antimicrobial Activity of Carvacrol. J. Agric. Food Chem..

[83] Ultee A., Kets E.P.W., Smid E.J. (1999). Mechanisms of Action of Carvacrol on the Food-Borne Pathogen *Bacillus cereus*. Appl. Environ. Microbiol..

[84] Alexopoulos A., Plessas S., Kimbaris A., Varvatou M., Mantzourani I., Fournomiti M. (2017). Mode of Antimicrobial Action of *Origanum vulgare* Essential Oil Against Clinical Pathogens. Curr. Res. Nutr. Food Sci..

[85] Friedman M. (2014). Chemistry and Multibeneficial Bioactivities of Carvacrol (4-isopropyl-2-methylphenol), a Component of Essential Oils Produced by Aromatic Plants and Spices. J. Agric. Food Chem..

[86] Lee J.-H., Kim Y.-G., Lee J. (2017). Carvacrol-Rich Oregano Oil and Thymol-Rich Thyme Red Oil Inhibit Biofilm Formation and the Virulence of Uropathogenic *Escherichia coli*. J. Appl. Microbiol..

[87] Pubchem Carvacrol. https://pubchem.ncbi.nlm.nih.gov/compound/carvacrol#section=Solubility.

[88] Koraichi Saad I., Hassan L., Ghizlane Z., Hind M., Adnane R. (2011). Carvacrol and Thymol Components Inhibiting Pseudomonas aeruginosa Adherence and Biofilm Formation. Afr. J. Microbiol. Res..

[89] Lambert R.J.W., Skandamis P.N., Coote P.J., Nychas G.-J.E. (2001). A Study of the Minimum Inhibitory Concentration and Mode of Action of Oregano Essential Oil, Thymol and Carvacrol. J. Appl. Microbiol..

[90] Ozkalp B., Sevgi F., Ozcan M., Ozcan M.M. (2010). The Antibacterial Activity of Essential Oil of Oregano (*Origanum vulgare* L.). J. Food Agric. Environ..

[91] De Falco E., Roscigno G., Landolfi S., Scandolera E., Senatore F. (2014). Growth, Essential Oil Characterization, and Antimicrobial Activity of Three Wild Biotypes of Oregano Under Cultivation Condition in Southern Italy. Ind. Crops Prod..

[92] Khosravi A.R., Shokri H., Kermani S., Dakhili M., Madani M., Parsa S. (2011). Antifungal Properties of *Artemisia sieberi* and *Origanum vulgare* Essential Oils Against *Candida glabrata* Isolates Obtained from Patients with Vulvovaginal Candidiasis. J. Mycol. Méd..

[93] Esen G., Azaz A.D., Kurkcuoglu M., Baser K.H.C., Tinmaz A. (2007). Essential Oil and Antimicrobial Activity of Wild and Cultivated *Origanum vulgare* L. subsp. *hirtum* (Link) Letswaart from the Marmara Region. Turk. Flavour Fragr. J..

[94] Burt S. (2004). Essential Oils: Their Antibacterial Properties and Potential Applications in Foods—A Review. Int. J. Food Microbiol..

[95] Zairi A., Nouir S., Khalifa M.A., Ouni B., Haddad H., Khelifa A., Trabelsi M. (2019). Phytochemical Analysis & Assessment of Biological Properties of Essential Oils Obtained from Thyme & Rosmarinus Species. Curr. Pharm. Biotechnol..

[96] Silva-Marrufo O., Marin-Tinoco R.I. (2021). Substitute of Synthetic Chemical Fungicides Using Oregano Essential Oil for Controlling *Fusarium oxysporum*. Gestion Ambiente.

[97] Servicio Meteorologico Nacional—National Metereological Service (SMN) (2010). Normales Climatologicas por Estado. Informacion Climatologica. Comision Nacional del Agua. https://smn.conagua.gob.mx/es/climatologia/informacion-climatologica/normales-climatologicas-por-estado.

[98] do Evangelho J.A., da Silva Dannenberg G., Biduski B., El Halal S.L.M., Kringel D.H., Gularte M.A., Fiorentini A.M., da Rosa Zavareze E. (2019). Antibacterial Activity, Optical, Mechanical, and Barrier Properties of Corn Starch Films Containing Orange Essential Oil. Carbohydr. Polym..

[99] Childs R.E., Bardsley W.G. (1975). The Steady-state Kinetics of Peroxidase with 2, 2′-azino-di-(3-ethyl-benzthiazoline-6-sulphonic acid) as Chromogen. Biochem. J..

[100] National Committee for Clinical Laboratory Standards (NCCLS) (2019). Methods for Dilution Antimicrobial Susceptibility Test for Bacteria That Grow Aerobically.

[101] Rossi C., Arias G., Lozano N. (2002). Evaluacion Antimicrobiana y Fitoquimica de *Lepechenia meyeni* Walp. Salvia. Cienc. Investig..

[102] Wiegand I., Hilpert K., Hancock R.E. (2008). Agar and Broth Dilution Methods to Determine the Minimal Inhibitory Concentration (MIC) of Antimicrobial Substances. Nat. Protoc..

[103] Buzzini P., Pieroni A. (2003). Antimicrobial Activity of Extracts of *Clematis vitalba* Towards Pathogenic Yeast and Yeast-Like Microorganisms. Fitoterapia.

[104] Jehl F., Bonnet R., Bru J., Caron F., Cattoen C., Cattoir V. (2016). Comité de L’antibiogramme de la Société Française de Microbiologie.

[105] Rivadeneira-Pacheco J.L., De La Hoz-Suarez A.I., Barrera-Argüello M.V. (2020). General Analysis of the SPSS and its Usefulness in Statistics. E-IDEA J. Bus. Sci..

